# Using rapid point-of-care tests to inform antibiotic choice to mitigate drug resistance in gonorrhoea

**DOI:** 10.2807/1560-7917.ES.2020.25.43.1900210

**Published:** 2020-10-29

**Authors:** Carolin Vegvari, Yonatan H Grad, Peter J White, Xavier Didelot, Lilith K Whittles, Nicole E Scangarella-Oman, Fanny S Mitrani-Gold, Etienne Dumont, Caroline R Perry, Kim Gilchrist, Mohammad Hossain, Tatum D Mortimer, Roy M Anderson, David Gardiner

**Affiliations:** 1 Department of Infectious Disease Epidemiology, School of Public Health, Imperial College London, London, United Kingdom; 2 Department of Immunology and Infectious Diseases, Harvard T.H. Chan School of Public Health, Boston, United States; 3 Division of Infectious Diseases, Brigham and Women’s Hospital, Harvard Medical School, Boston, United States; 4 MRC Centre for Global Infectious Disease Analysis and NIHR Health Protection Research Unit in Modelling and Health Economics, School of Public Health, Imperial College London, London, United Kingdom; 5 Modelling and Economics Unit, National Infection Service, Public Health England, London, United Kingdom; 6 Current affiliation: School of Life Sciences and Department of Statistics, University of Warwick, United Kingdom; 7 GlaxoSmithKline, Collegeville, Pennsylvania, United States; 8 Current affiliation: Pfizer, Inc, Pennsylvania, United States

**Keywords:** antibiotic resistance, gonorrhoea, point-of-care tests, sexually transmitted infections, bacterial infections, gonorrhoea, antibiotic use, antimicrobial resistance, multidrug resistance, epidemiology, modelling, molecular methods

## Abstract

**Background:**

The first cases of extensively drug resistant gonorrhoea were recorded in the United Kingdom in 2018. There is a public health need for strategies on how to deploy existing and novel antibiotics to minimise the risk of resistance development. As rapid point-of-care tests (POCTs) to predict susceptibility are coming to clinical use, coupling the introduction of an antibiotic with diagnostics that can slow resistance emergence may offer a novel paradigm for maximising antibiotic benefits. Gepotidacin is a novel antibiotic with known resistance and resistance-predisposing mutations. In particular, a mutation that confers resistance to ciprofloxacin acts as the ‘stepping-stone’ mutation to gepotidacin resistance.

**Aim:**

To investigate how POCTs detecting *Neisseria gonorrhoeae* resistance mutations for ciprofloxacin and gepotidacin can be used to minimise the risk of resistance development to gepotidacin.

**Methods:**

We use individual-based stochastic simulations to formally investigate the aim.

**Results:**

The level of testing needed to reduce the risk of resistance development depends on the mutation rate under treatment and the prevalence of stepping-stone mutations. A POCT is most effective if the mutation rate under antibiotic treatment is no more than two orders of magnitude above the mutation rate without treatment and the prevalence of stepping-stone mutations is 1–13%.

**Conclusion:**

Mutation frequencies and rates should be considered when estimating the POCT usage required to reduce the risk of resistance development in a given population. Molecular POCTs for resistance mutations and stepping-stone mutations to resistance are likely to become important tools in antibiotic stewardship.

## Introduction

*Neisseria gonorrhoeae*, the causal agent of the sexually transmitted infection (STI) gonorrhoea, is becoming increasingly resistant to available antibiotic treatment options [1,2]. The most widely recommended treatment for gonorrhoea is a combination therapy of ceftriaxone plus azithromycin, administered empirically without bacterial culture or point-of-care testing [3]. In isolates collected across Europe, the proportion of isolates with decreased susceptibility to ceftriaxone increased from 15% to 17.7% from 2015 to 2016. At the same time azithromycin resistance across Europe was stable at about 7% but was much higher in individual countries (34.5% in Portugal) [4]. The first treatment failure of this dual therapy was reported in the United Kingdom (UK) in 2014 [5]. Azithromycin resistance in combination with reduced susceptibility to ceftriaxone has been well-studied [4]. Resistance to previous recommended treatments, such as ciprofloxacin, is generally high (30-70% in Europe, above 70% in East Asia) [2]. As ceftriaxone is at the same time the first-line and last-resort treatment, the World Health Organization (WHO) in 2017 declared the possible evolution of untreatable gonorrhoea a global public health emergency [6].

In an attempt to spare ceftriaxone as a last-resort treatment, rapid point-of-care tests (POCTs) detecting ciprofloxacin resistance mutations have been developed. Thus, even though ciprofloxacin is no longer recommended for gonorrhoea treatment, it could still be used when a POCT detects no resistance mutations [7]. Such tests could easily be expanded to include known resistance markers for other antibiotics.

Gepotidacin is a novel topoisomerase IIA inhibitor currently under development and in phase III clinical trials with activity against *N. gonorrhoeae* [8]. Its mechanism of action differs from that of fluoroquinolones, and it has demonstrated activity against most ciprofloxacin-resistant gonococcal strains [9]. Ciprofloxacin inhibits bacterial DNA gyrase and topoisomerase IV. The main ciprofloxacin resistance mutations in genes coding for DNA gyrase subunit A (GyrA) and topoisomerase IV subunit A (ParC) in *N. gonorrhoeae* are presented in the Supplementary Material 1, Supplementary Table 1. In a recent phase II clinical trial on the efficacy of gepotidacin against uncomplicated genitourinary gonorrhoea, emergence of resistance was observed for *N. gonorrhoeae* isolates from two treatment failures following use of a single dose of 3g gepotidacin. This resistance is likely to have emerged due to the combination of a pre-existing ciprofloxacin resistance mutation (D86N) in the *parC* gene and de novo within-host emergence of an A92T mutation in the *gyrA* gene (Table 1). Additional experiments suggest that the gepotidacin minimum inhibitory concentration (MIC) is only significantly increased if both mutations are present together [10]. See Supplementary Material 1, Supplementary Text 1 for more details on the microbiological analysis of the phase II clinical trial. Structural analysis of the interaction of gepotidacin with GyrA suggests that gepotidacin does not interact with the two quinolone binding sites in GyrA at amino acid positions 91 and 95 [9]. Therefore, it was assumed that the S91F and D95G mutations in *gyrA* were not critical for the evolution of gepotidacin resistance. There may be other mutations that can cause resistance to gepotidacin, but they were not observed in the phase II clinical trial.

**Table 1:** Genotypes of isolates at baseline and test-of-cure from gepotidacin treatment failures with emergence of resistance, phase II clinical trial, 2017 [11]

Participant number	Visit	Genotype *gyrA*	Genotype *parC*	MIC gepotidacin (mg/L)	MIC ciprofloxacin (mg/L)
4	Baseline	S91F D95G	D86N	1	8
	Test-of-cure	S91F **A92T** D95G	D86N	> 32	8
6	Baseline	S91F D95G	D86N	1	4
	Test-of-cure	S91F **A92T** D95G	D86N	32	4

MIC: minimum inhibitory concentration.

Mutation *gyrA* A92T leading to gepotidacin resistance is displayed in bold.

Novel post-treatment mutations occurred in isolates from two subjects that were treated with a single dose of 3g gepotidacin [11]. The mutations S91F and D95G in *gyrA* and D86N in *parC* on their own confer different levels of resistance to ciprofloxacin.

Here, we consider the novel paradigm of introducing an antibiotic together with a POCT to control gonococcal infections and slow down resistance development. A POCT for gepotidacin resistance would determine if the known stepping-stone mutations, *gyrA* A92T or *parC* D86N, were present. If neither were detected, then gepotidacin could be used without substantial risk of treatment failure, based on current evidence, as there are no other known clinically relevant target-specific resistance mutations for gepotidacin in *N. gonorrhoeae*. If one or both mutations were present, treatment with another antibiotic would be indicated.

Determining the frequencies of resistance mutations requires surveillance systems such as the data recorded in the European Gonococcal Antimicrobial Surveillance Programme (Euro-GASP, data collection since 2009) and the US Gonococcal Isolate Surveillance Project database (GISP, data collection since 1986). Euro-GASP monitors *N. gonorrhoeae* antimicrobial susceptibility trends by phenotypically characterising isolates from male and female patients. GISP samples isolates from male patients attending STD clinics. Each participating country contributes 100 cultured and characterised isolates per year.

Our study aims to answer several questions using a theoretical modelling framework: can a molecular POCT that detects known stepping-stone mutations prevent the spread of gepotidacin-resistant strains? Under what conditions is a POCT most effective at reducing the risk of resistance development, and how frequently would such a test need to be used to reduce this risk by at least 50% over five years? These questions have broader implications for designing antibiotic stewardship strategies and prolonging the life span of novel and existing antibiotics.

## Methods

### Model framework

**Figure 1:**
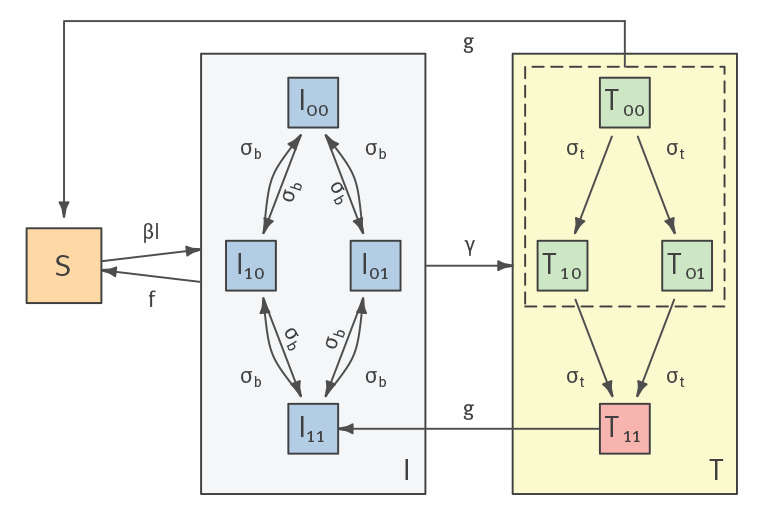
Two-locus gonorrhoea antibiotic resistance model

We developed a compartmental deterministic model framework of gonorrhoea transmission building on previous models [12,13]. As in Whittles et al. [14] our model uses transmission parameter values derived from men who have sex with men (MSM) populations in London. The model has three compartments, susceptible (S), infected (I) and treated (T) individuals (Figure 1, Supplementary Material 1, Supplementary Text 2). Individuals in the infected class are infected but not currently treated. Individuals in the treated class are infected and currently receiving treatment. The time that individuals spend in the treated class is the duration for which the within-host antibiotic concentration is great enough to clear the infection.

S: susceptible individuals; I: infected, untreated individuals; T: infected, treated individuals.

The basic model has three main compartments, susceptible, infected and treated individuals (i.e. infected individuals that are undergoing treatment). Individuals can be infected with one of four gonococcal strains that differ in the combination of resistance (‘stepping-stone’) mutations they carry (00, 10, 01 or 11). Only strains with both gepotidacin resistance mutations (11) are considered resistant to gepotidacin. Strains with one resistance mutation (10 and 01) are resistant to ciprofloxacin, but sensitive to gepotidacin. First subscript: state of resistance for the first locus (0 – sensitive, 1 – resistant); second subscript: state of resistance for the second locus (0 – sensitive, 1 – resistant); β: infection rate parameter (product of sexual contact rate and infection probability per contact); f: spontaneous recovery rate from infection without treatment (1/f – duration of natural infection); g: Cure rate in individuals currently being treated (1/g – duration of treatment); γ: treatment seeking rate (1/γ – time from start of infection to start of treatment); σ_b_: locus-specific mutation rate in untreated individuals (no selection pressure); σ_t_: locus-specific mutation rate in treated individuals (under selection pressure).

The key novel feature of our model is that we explicitly consider different resistance genotypes of relevance to gepotidacin. Given that there are two known stepping-stone mutations that together cause elevated MICs, susceptible individuals can be infected by one of four strains. If 0 signifies the sensitive allele and 1 the resistant allele, these strains are: 00, 10, 01 and 11. Based on the observations from the phase II clinical study [15], we assume that only the 11 genotype is resistant to both ciprofloxacin and gepotidacin, whereas 10 and 01 are resistant to ciprofloxacin only. The model also allows for the possibility that both resistance mutations can arise de novo over the course of an infection.

### Model parameters and transitions

Susceptible individuals become infected at rate β, which in our modelling framework is population-specific and depends on the sexual contact rate and the infection probability per contact (Table 2). Infected individuals seek treatment at rate γ and can recover spontaneously at rate f. Treated individuals recover at rate g if they are not resistant, and return to the susceptible class. Individuals with treatment failures are reclassified as infected. For more details on the model processes see Supplementary Material 1, Supplementary Text 2. To assess the impact of uncertainty in model parameters, we performed sensitivity analyses by varying model parameters across a range of measured values from the literature. We assume an annual incidence rate of gonorrhoea of 22,000 cases in a total population of 1.5 million individuals, approximating the MSM population in the UK [16]. We varied the starting conditions for each of the simulation scenarios described below. For model parameter and variable values used in the simulations see Table 2.

**Table 2:** Parameter values used in simulation model

Model parameter (unit)	Values used in individual simulations
Infection rate (per day)	5.56 × 10 ^− 8^, 1.67 × 10 ^− 8^, 6.02 × 10 ^− 8^, 2.28 × 10 ^− 7^, 2.29 × 10 ^− 7^
Recovery rate f (inverse of duration of natural infection) (per day)	1/84, 1/160, 1/185, 1/240, 1/365
Treatment rate γ (inverse of time in days until patients first seek treatment) (per day)	1/3, 1/12, 1/13, 1/52
Cure rate for gepotidacin treatment, assuming double dose (inverse of treatment duration, i.e. time over MIC) (per day)	1.778 ( = 1/13.5h)
Cure rate for ciprofloxacin treatment, assuming single dose (inverse of treatment duration) (per day)	6 ( = 1/4h)
Proportion of patients that return for second round treatment p	1, 0.8, 0.6, 0.5
Mutation rate without treatment σ_b_ (substitutions per nt per day)	3.12 × 10 ^− 9^, 2.45 × 10 ^− 8^
Mutation rate with treatment σ_t_ (substitutions per nt per day)	3.12 × 10 ^− 9^, 2.45 × 10 ^− 8^, 4.9 × 10 ^− 8^, 1.23 × 10 ^− 7^, 2.45 × 10 ^− 7^, 2.45 × 10 ^− 6^, 2.45 × 10 ^− 5^, 7.95 × 10 ^− 5^, 9.66 × 10 ^− 4^
Point-of-care test usage (%)	0, 10, 20, 30, 40, 50, 60, 70, 80, 90, 100
Total simulated population	1.5 × 10^6^
Initial number of infected individuals/equilibrium incidence rate	22,000
Initial prevalence of *parC* D86N (%)	0, 0.06, 0.18, 0.462, 0.669, 1.5, 2, 2.9, 3, 5.9, 6.5, 8.6, 13, 19.3, 38.6
Initial prevalence of *gyrA* A92T (%)	0, 1
Initial prevalence of double mutant (*parC* D86N/*gyrA* A92T) (%)	0

MIC: minimum inhibitory concentration.

All rates are per day. If more than one value is given, the whole range of values has been tested in different simulations. See Supplementary Material 2 for parameter combinations used in individual simulations. References and the basis of assumptions are included in the Supplementary Material 1, Supplementary Table 2.

### Resistance evolution

Sensitive strains can acquire resistance to antibiotics by de novo mutations. The mutation rate in *N. gonorrhoeae* has been determined from phylogenetic studies [17]. Several studies indicate that the mutation rate under treatment may be increased due to the SOS DNA damage response [18-20]. A DNA damage response system in *N. gonorrhoeae* has been described by Schook et al. [21]. Other topoisomerase II inhibitors are known to increase the mutation rate by interfering with DNA replication [22,23]. However, as the mechanism of action of gepotidacin differs from that of conventional topoisomerase II inhibitors, it may not increase the mutation rate to the same extent. There are no estimates for mutation rates in *N. gonorrhoeae* under antibiotic pressure. We therefore performed simulations for a range of mutation rate parameters under treatment based on estimates obtained from other bacterial species (Table 2).

*N. gonorrhoeae* is known to have a high rate of homologous recombination [24]. Recombination between different gonococcal strains can only occur in mixed infections at the same anatomical site. Thus, the effective recombination rate can be calculated as:

coinfection frequency × ratio of recombination to mutation × base mutation rate

The coinfection frequency with different gonococcal strains at the same anatomical site is unknown, but we can use the frequency of infections with different gonococcal strains at different anatomical sites as a proxy upper-bound estimate for the frequency of mixed infections (13%) at the same anatomical site [25]. The ratio of recombination to mutation events has been estimated from whole genome sequence data (genome-wide average) [17,26,27]. If we assume a mutation rate of 2.45 × 10 ^− 8^ substitutions per nt per day and a recombination-to-mutation ratio of 2.2 [27], we obtain an effective recombination rate of 7 × 10 ^− 9^. Since this would lead to an increase in the rate of resistance acquisition that is smaller than the increased mutation rates that we tested, we do not explicitly consider recombination in the model.

### Treatment scenarios and outcome measure

In our numerical evaluations of the model of a POCT detecting resistance mutations we varied the use of the POCT as a proportion of treated gonorrhoea infections from 0% to 100% and the assumed sensitivity and specificity of the test from 80 to 100%. If a POCT was used then gepotidacin was only used as a treatment if no resistance mutations were detected. If no POCT was used then gepotidacin was used as a first-line treatment.

We used stochastic simulations based on the deterministic structure defined in Supplementary Material 1, Supplementary Text 2, Figure S1, Equations 2, Table S1, and a Gillespie algorithm to analyse model behaviour and predictions. We recorded the number of simulations out of 100 replications in which the 5% resistance threshold was reached at any time point over a five-year timeframe. (This corresponds to the WHO recommendation that when resistance to a specific antibiotic exceeds 5%, alternative antibiotics should be used [28].) Table 2 lists parameter values used in simulations. A full list of parameter combinations used in each simulation scenario together with the results can be found in Supplementary Material 2.

### Determining the prevalence of *parC* D86N in Europe and the United States

We obtained publicly available *N. gonorrhoeae* whole genome sequencing (WGS) data from the National Center for Biotechnology Information (NCBI) Sequence Read Archive deposited as part of the studies in Supplementary Material 1, Supplementary Table 3. We ran FastQC [29] to assess WGS data quality and removed accessions with insufficient or poor-quality reads. We mapped reads to *N. gonorrhoeae* NCCP11945 (NC_011035.1) using BWA-MEM vs 0.7.17-r1188 [30]. Duplicates were marked using Picard vs 2.8.0 (https://github.com/broadinstitute/picard). We called variants using Pilon vs 1.23 [31] with minimum depth of 10X and minimum mapping quality of 20. We removed accessions where more than 15% of sites were unable to be called by Pilon due to insufficient coverage or poor mapping quality. We identified variants in *gyrA* and *parC* corresponding to the amino acid mutations *gyrA* A92T and *parC* D86N.

Currently, no published genomic databases report frequencies for the *gyrA* A92T mutation. The highest reported prevalence of the *parC* D86N mutation was 38.6% of ciprofloxacin-resistant isolates [12]. We genotyped 10,259 unique accessions that passed our quality control filters. The frequencies of *parC* D86N and *gyrA* A92T mutations are low in Europe and in the United States (Euro-GASP: *parC* D86N 1.8%, *gyrA* A92T 0% of all gonococcal isolates analysed September–November 2013 [32], GISP: *parC* D86N 0.635%, *gyrA* A92T 0%, of all gonococcal isolates analysed 2000-2013 [33]). This means that in Europe and the US simulation assuming 0.6–6.5% initial prevalence of *parC* D86N are the most applicable.

### Ethical statement

No ethical approval was required for this study because no new data have been collected as part of the study.

## Results

**Figure 2:**
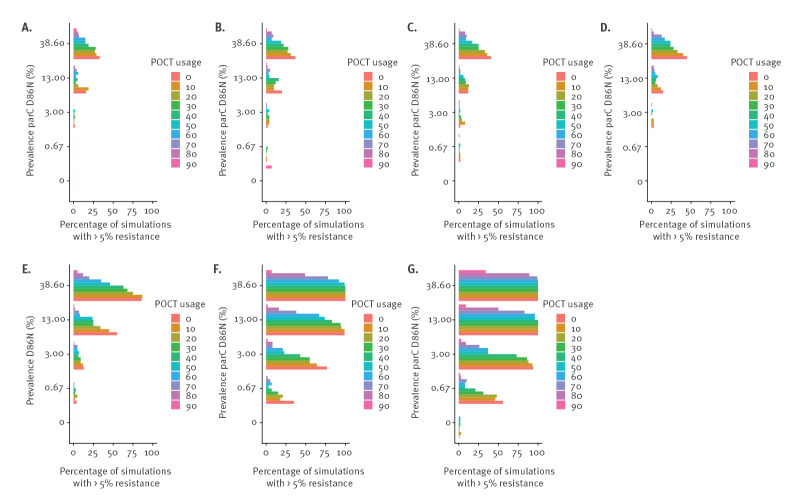
Proportion of simulations in which the frequency of gepotidacin-resistant strains reaches 5% with different mutation rates, prevalence of *parC* D86N and POCT usage levels

If the mutation rate with treatment is the same as without treatment, then even a POCT usage of 20–30% can reduce the risk of resistance development (Figure 2A). With assumptions of complete testing and perfect sensitivity and specificity, resistance did not develop in our simulations. If the initial prevalence of the stepping-stone mutations was lower than 6%, stochastic effects were important, so that even high POCT usage had little impact on the emergence of resistant strains (Supplementary Material 1, Supplementary Figure 1). In populations with an initial frequency of more than 6% of the *parC* D86N mutation, a POCT had a potential to reduce the risk of resistance reaching 5%. The effect of the POCT was roughly proportional to the usage level.

POCT: point-of-care testing.

The initial prevalence of *gyrA* A92T was 0 in all simulations. Mutation rates (substitutions per nt per day): A. 2.45 × 10 ^− 8^, B. 4.9 × 10 ^− 8^, C. 1.23 × 10 ^− 7^, D. 2.45 × 10 ^− 7^, E. 2.45 × 10 ^− 6^, F. 2.45 × 10 ^− 5^, G. 7.95 × 10 ^− 5^. Total number of simulations 100.

The greater the mutation rate during treatment and the higher the population prevalence of stepping-stone mutations, the higher the POCT usage needed to reduce the risk of resistance development (Figure 2). With an initial frequency of the *parC* D86N greater than 10% and a mutation rate during treatment of more than 1,000 times the baseline mutation rate, POCT usage had to be 80–90% to halve the risk of resistance development (Figure 2G). An increase in mutation rate during treatment of this order of magnitude is rarely observed in laboratory experiments (Supplementary Material 1, Supplementary Table 4). If the initial prevalence of *gyrA* A92T is 1%, rather than 0%, more resistance emerges, especially if mutation rates under treatment are high (Supplementary Material 1, Supplementary Figure 2). If the mutation rate under treatment is less than 1,000 times the baseline mutation rate, the added risk of resistance development from *gyrA* A92T prevalence of 1% stays below 10%.

If there were no stepping-stone mutations in a population, the risk of resistance development was generally low (< 5% if the prevalence of *parC* D86N was < 13% and only exceeding 25% if the prevalence of *parC* D86N was 38.6%), and a POCT was only required if the mutation rate during treatment was very high (9.66 × 10^-4^ per site per day) (Supplementary Material 1, Supplementary Figures 1, 3). The sensitivity and specificity of the test did not have a great influence on the risk of resistance development in the range tested. With high POCT usage (70–90%), a higher sensitivity (99% compared to 80%) of the test slightly decreases the probability of resistance spreading (Supplementary Material 1, Supplementary Figure 4).

### *parC* D86N prevalence

We did not observe any isolates with *gyrA* A92T. We found that across all datasets 6.1% (626/10,259) of isolates encoded the *parC* D86N mutation. *parC* D86N was observed in 17 of 20 datasets.

If the mutation rate is not increased under treatment, the risk of resistance emergence is less than 5% in scenarios assuming 0.6 – 6.5% initial prevalence of *parC* D86N.

## Discussion

Our results indicate that a molecular POCT detecting the two known stepping-stone mutations implicated in gepotidacin resistance could help reduce the risk of resistance development to gepotidacin, a novel antibiotic undergoing phase III trials, by *N. gonorrhoeae*. The ability to do so would depend on the population prevalence of stepping-stone mutations and the mutation rate under treatment. If both are low, then most strains will be sensitive to gepotidacin and a POCT would have a negligible effect on the risk of resistance development. If the mutation rate under treatment is very high and a large proportion of strains already have one stepping-stone mutation, a POCT would not be able to prevent resistance spreading, because resistance would arise too frequently after testing in previously sensitive infections. A high rate of horizontal gene transfer between coinfecting strains could equally lead to increased rates of resistance emergence [34]. It is possible that other fluoroquinolone resistance mutations affect the MIC for gepotidacin in *N. gonorrhoeae*, but none have so far been identified.

This suggests that a POCT would be most valuable if the increase in mutation rate under treatment is moderate (no more than 100 times above the baseline mutation rate) and the prevalence of pre-existing resistance mutations is at least 1%. In this case and if the prevalence of resistance mutations is not too high (maximum 13% in our analyses), even a 20–30% usage of the POCT could, given our assumptions, halve the risk of resistance development. This would be the case for all publicly available datasets we surveyed, where 6.1% of all *N. gonorrhoeae* genomes carry *par*C D86N. However, due to stochastic variability, 50% usage would be preferable to reliably halve the risk of resistance development. The prevalence of *parC* D86N is expected to vary among different countries. Therefore, optimum POCT usage values will be country-specific.

These results are in good agreement with a recent study which found that a POCT that detects resistance to three antibiotics used to treat gonorrhoea can prevent resistant strains from spreading, if its usage is at least 37%, and that test sensitivity and specificity have a minor effect on resistance development [35]. Our study also agrees with results from Fingerhuth et al. according to which a POCT test with resistance detection prevents more cases of antibiotic-resistant gonorrhoea than a NAAT test without resistance detection, unless the POCT sensitivity is lower than 80% [36].

Since gepotidacin resistance only arises when both known stepping-stone mutations occur in the same strain, the relationship between the mutation rate under treatment and the risk of resistance development is not linear. Small increases in mutation rate of up to 10-fold did not increase the risk of resistance development in our simulations, unless the initial prevalence of *parC* D86N was assumed to be greater than 30%. If the mutation rate under treatment increased 1,000 times or more, resistance almost always developed within 5 years.

Mutation rates during antibiotic exposure of this magnitude are rare according to the literature on other bacterial species (Supplementary Material 1, Supplementary Table 4). Moreover, mutation rate measurements from in vitro experiments are prone to overestimation [37]. Our results suggest that estimates of the mutation rate under antibiotic exposure should be taken into account when evaluating treatment strategies. For example, Obolski and Hadany use a simulation model to show that in hospitals antibiotic mixing and cycling are superior to combination therapy, if bacterial mutagenesis is stress-induced [38].

We did not consider fitness costs of antibiotic resistance mutations, because there is no population-level data on potential fitness costs of gepotidacin resistance mutations. As fluoroquinolone-resistant strains persist in the population, we can assume that fitness costs associated with fluoroquinolone-resistance mutations are small or absent [33]. Our model represents a worst-case scenario regarding the speed of spread of gepotidacin resistance. If there were sufficiently high fitness costs associated with one or both known stepping-stone mutations leading to gepotidacin resistance and a POCT could ensure that only infections without stepping-stone mutations were treated with gepotidacin, then newly-arising gepotidacin-resistant strains would potentially quickly become extinct [14].

Since some of the data for this study came from a relatively small sample (a phase II clinical trial), the evaluation may have to be updated when more data becomes available. In the case of treatment failure, the sequence in which alternative antibiotics are prescribed can matter, especially if there is evidence for cross-resistance or resistance mutations to different antibiotics for the same strains. Therapies with multiple targets, or antibiotics that require multiple mutations before they lose their efficacy, should be preferred as first-line treatments.

The main limitation of this study is the lack of empirical information on key model parameters. For example, estimates for the duration of natural infection are based on limited observational studies from before 1980. Similarly, the duration from infection to when patients seek treatment may vary among different populations. The population we model approximates an MSM population and likely overestimates treatment rates for women who are more frequently asymptomatic. However, as long as we compare simulation scenarios with the same sets of parameters, the qualitative outcome of our analysis is unlikely to change. Another limitation is that potentially we do not know all mechanisms of resistance to gepotidacin and we acknowledge the need for genomic surveillance to determine if other resistance mutations can arise.

Simulation studies can inform us on what data should be collected to improve treatment strategies. In the case of gepotidacin, molecular surveillance data to estimate the frequency of known stepping-stone mutations is required. More generally, whole-genome surveillance data in combination with phenotypic antimicrobial susceptibility data can inform us about the frequency of resistance genes to other antimicrobials used for gonorrhoea treatment. In vitro or animal model experiments could help to estimate the mutation rate under gepotidacin exposure. Mutation prevalence and rate should be considered when estimating the POCT usage required to reduce the risk of resistance development in a given population. Molecular POCTs for resistance mutations and stepping-stone mutations are likely to become important tools in antibiotic stewardship and surveillance in the coming years, and a combination of empirical study and modelling is required to optimise their use for public health benefit.

## Supplementary Data

1397000 Supplementary Material 1

## Supplementary Data

201000 Supplementary Material 2
