# Erratum for Euro Surveill. 2020;25(43)

**DOI:** 10.2807/1560-7917.ES.2020.25.45.2011127

**Published:** 2020-11-12

**Authors:** Eurosurveillance editorial team

**Affiliations:** 1 European Centre for Disease Prevention and Control (ECDC), Stockholm, Sweden

In the article ‘Using rapid point-of-care tests to inform antibiotic choice to mitigate drug resistance in gonorrhoea’ by Vegvari et al., published on 29 Oct 2020, the affiliation of Fanny S Mitrani-Gold was corrected after publication of the article, on 6 Nov 2020.

