# Supplementary material for: Using rapid point-of-care tests to inform antibiotic choice to mitigate drug resistance in gonorrhoea
Source: Euro Surveill. 2020 Oct 29;25(43):1900210. doi: 10.2807/1560-7917.ES.2020.25.43.1900210 (PMC7596916; doi:10.2807/1560-7917.ES.2020.25.43.1900210)
Supplement: Supplementary Material 1 [file 1900210_Supplementary_Material_1.pdf]

Disclaimer: This supplementary material is hosted by *Eurosurveillance* as supporting information alongside the article 'Using rapid point-of-care tests to inform antibiotic choice to mitigate drug resistance in gonorrhoea', on behalf of the authors, who remain responsible for the accuracy and appropriateness of the content. The same standards for ethics, copyright, attributions and permissions as for the article apply. Supplements are not edited by *Eurosurveillance* and the journal is not responsible for the maintenance of any links or email addresses provided therein.

Supplementary Table 1: Main ciprofloxacin resistance mutations in the target genes in *Neisseria gonorrhoeae* (1). Changes in the amino acid positions increase ciprofloxacin MICs against *N. gonorrhoeae*. The first letter (e.g. S) is the wildtype amino acid and the # (e.g. 91) is the amino acid position at which a change occurs.

Target	Resistance mutations/amino acid changes
GyrA	S91[±D95][1]
ParC	D86, S87, S88, E91

[1] The combination of S91 and D95 confers a greater level of resistance to ciprofloxacin (measured in terms of MIC) than either mutation alone (2).

Supplementary Text 1 – Microbiological Analysis of the gepotidacin phase II trial

Five baseline isolates in the phase II clinical trial had a gepotidacin MIC of 1 mg/L and were ciprofloxacin-resistant, compared to a modal gepotidacin MIC of 0.5 mg/L for ciprofloxacin-resistant isolates in a separate study, and an MIC of 32 mg/L for test-of-cure isolates from two of three treatment failures in the phase II clinical trial (3, 4). The ratio of the area under the free-drug concentration-time curve to the MIC (fAUC/MIC) was associated with microbiologic success in the trial. Success was 100% at fAUC/MICs of ≥48 and decreased to 63% for fAUC/MICs of ≤25. All 3 isolates from microbiological failures were ciprofloxacin-resistant, had a baseline gepotidacin MIC of 1 mg/L, and carried a ParC D86N mutation, a critical residue for gepotidacin binding. Test-of-cure isolates from two of the three treatment failures demonstrated resistance emergence to gepotidacin with MICs of ≥32mg/L and had an additional GyrA A92T mutation, also a critical residue for gepotidacin binding. Therefore, the ParC D86N (first step) and GyrA A92T (second step) mutations can be considered 'stepping-stone' mutations with respect to gepotidacin resistance.

Supplementary Table 2. All rates are per day. If more than one value is given, the whole range of values has been tested in different simulations. See Supplementary Excel workbook for parameter combinations used in individual simulations.

Model parameter [unit]	Values used in individual simulations	References
Infection rate parameter [per day]	5.56×10^{-8}, 1.67×10^{-8}, 6.02×10^{-8}, 2.28×10^{-7}, 2.29×10^{-7}	Fitted so that the equilibrium annual incidence rate in the absence of resistant strains was about 22,000 (total population $1.5\text{x}10^{6}$) (5)
Recovery rate f (inverse of duration of natural infection) [per day]	1/84, 1/160, 1/185, 1/240, 1/365	(6-12)
Treatment rate γ (inverse of time in days until patients first seek treatment) [per day]	1/3, 1/12, 1/13, 1/52	(9, 13, 14)
Cure rate gepotidacin, assuming double dose (inverse of treatment duration, i.e. time over MIC) [per day]	1.778 (=1/13.5h)	Information provided by GSK (derived from simulations of PK model published in (15, 16))
Cure rate ciprofloxacin, assuming single dose (inverse of treatment duration) [per day]	6 (=1/4h)	(17)
Proportion of patients that return for 2nd round treatment p	1, 0.8, 0.6, 0.5	Assumptions (1 means all patients return for 2nd round treatment, but because this perfect scenario is unlikely, we tested several lower values)
Mutation rate without treatment σ_b [substitutions per nucleotide per day]	3.12×10^{-9}, 2.45×10^{-8}	(12, 18, 19)
Mutation rate with treatment σ_t [substitutions per nucleotide per day]	3.12×10^{-9}, 2.45×10^{-8}, 4.9×10^{-8}, 1.23×10^{-7}, 2.45×10^{-7}, 2.45×10^{-6}, 2.45×10^{-5}, 7.95×10^{-5}, 9.66×10^{-4}	Assumptions (based on mutation rates published for bacteria under antibiotic treatment: (20-28))
Point-of-care test usage [%]	0, 10, 20, 30, 40, 50, 60, 70, 80, 90, 100	Assumptions (sensitivity analysis over the possible range of POCT uptake from 0-100%)
Total simulated population	1.5×10^{6}	(5)
Initial number of infected individuals/equilibrium incidence rate	22,000	(5)
Initial prevalence of ParC D86N [%]	0, 0.06, 0.18, 0.462, 0.669, 1.5, 2, 2.9, 3, 5.9, 6.5, 8.6, 13, 19.3, 38.6	Assumptions based on multiplying published frequencies of ParC D86N mutations in quinolone-resistant strains with published levels of

		ciprofloxacin resistance (29-37)
Initial prevalence of GyrA A92T [%]	0, 1	(29-33, 38)
Initial prevalence of double mutant (ParC D86N/GyrA A92T) [%]	0	Assumption, not reported in any dataset

Supplementary Table 3. Whole genome sequencing data sets and prevalence of ParC D86N.

Study	Country	Proportion of Isolates with ParC D86N
Grad et al. 2014 (18), Grad et al. 2016 (39)	United States	0.00636 (7/1100)
Ezewudo et al. 2015 (19)	Global	0.0185 (1/54)
Demczuk et al. 2015 (40)	Canada	0.00840 (1/119)
Demczuk et al. 2016 (41)	Canada	0.0196 (4/204)
De Silva et al. 2016 (42)	United Kingdom	0.0215 (40/1823)
Eyre et al. 2017 (43)	United Kingdom	0.0476 (22/462)
Kwong et al. 2017 (44)	Australia	0.0851 (8/94)
Buckley et al. 2018 (45)	Australia	0 (0/372)
Cehovin et al. 2018 (46)	Kenya	0 (0/103)
Fifer et al. 2018 (47)	United Kingdom	0 (0/50)
Harris et al. 2018 (48)	Europe	0.0442 (46/1041)
Lee et al. 2018 (49)	New Zealand	0.0242 (10/297)
Ryan et al. 2018 (50)	Ireland	0.179 (7/39)
Sánchez-Busó et al. 2018 (51, 52)	Global	0.0548 (21/383)
Yahara et al. 2018 (53)	Japan	0.0923 (24/260)
Peng et al. 2019 (54)	China	0.144 (60/416)
Thomas et al. 2019 (55)	United States	0.0946 (58/613)
Williamson et al. 2019 (56)	Australia	0.114 (249/2181)
Lan et al. 2020 (57)	Vietnam	0.0617 (14/227)
Town et al. 2018 (58)	United Kingdom	0.0691 (88/1274)

Supplementary Table 4: Examples of mutation rate increases with treatment

Bacterial species	Antibiotic	Mutation rate or relative increase	Reference
Mycobacterium fortuitum	Ciprofloxacin 0.5MIC	72-120-fold increase	(21)
Streptococcus pneumoniae	Ciprofloxacin 2MIC	1st step 1.1×10^{-9} per nucleotide per replication<br>2nd step 1.3×10^{-8} per nucleotide per replication	(22)
	Gemifloxacin	1st step 1.6×10^{-11} per nucleotide per replication<br>2nd step 7.2×10^{-9} per nucleotide per replication	(22)
Pseudomonas aeruginosa	Levofloxacin	0 to >1 in 74000 colonies depending on strain and concentration	(20)
	Ciprofloxacin	0 to >1 in 80000 colonies depending on strain and concentration	(20)
	Quinolone	Frequency of resistant colonies 1.2×10^{-6} to 4×10^{-10} depending on quinolone and concentration	(59)
	Ceftazidime	200-fold increase in mutant cells	(28)
	Ciprofloxacin	50000-fold increase in mutant cells	(28)
	Tobramycin	10-fold increase in mutant cells	(28)
Campylobacter jejuni	Ciprofloxacin 10MIC	≥1.5-fold increase	(23)
Escherichia coli	Norfloxacin	Concentration- and strain-dependent, in the order of 1×10^{-10} to 1×10^{-8} per nucleotide per cell division	(24)
	Ciprofloxacin	Wild-type 7-fold increase<br>Hypoactive mutator strain 1.3-fold increase<br>Hyperactive mutator strain 13-fold increase	(60)
S. aureus	Ciprofloxacin	4.3-fold increase	(27)
	Vancomycin	1.7-2.5-fold increase	(27)

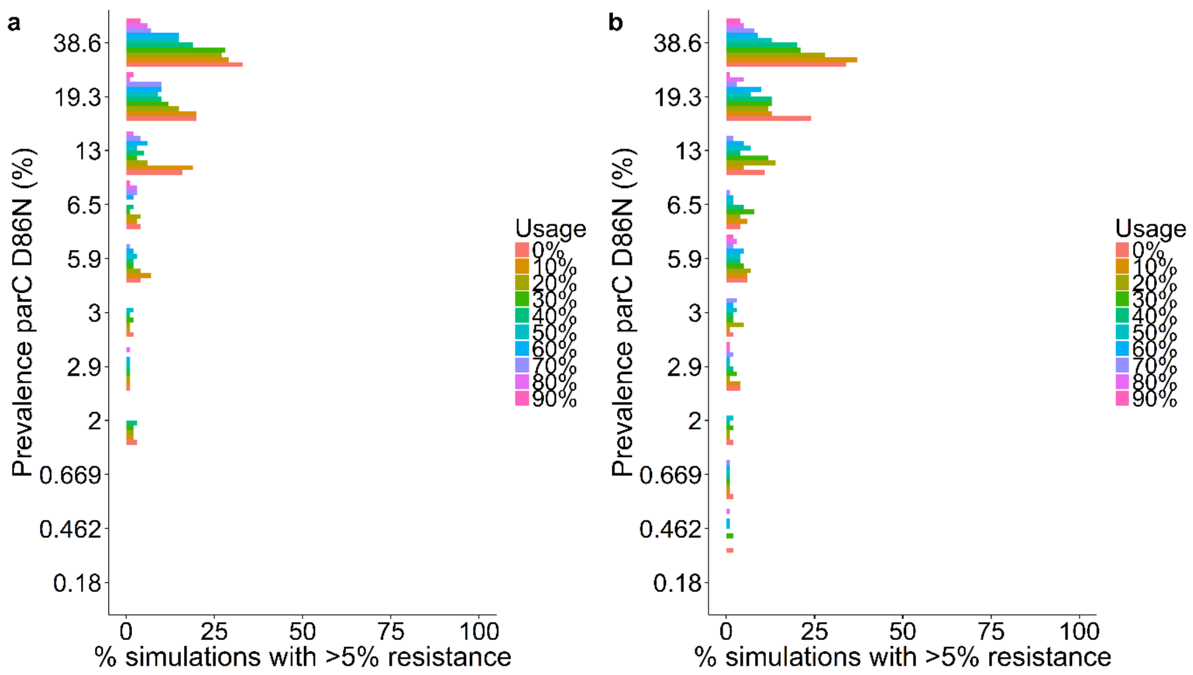

Supplementary Figure 1: Proportion of simulations in which the frequency of gepotidacin-resistant strains reaches 5% with different stepping-stone mutation prevalences and POCT usage levels. The mutation rate with treatment is assumed to be the same as without treatment, 2.45×10^{-8} substitutions per nucleotide per day. a) Prevalence of GyrA A92T is 0. b) Prevalence of GyrA A92T is 1%. There was one simulation in which resistance occurred if mutation 1 had a frequency of 0 and POCT usage was 0%. X-axis: Percentage of simulations in which 5% gepotidacin resistance is reached. Y-axis: prevalence of ParC D86N.

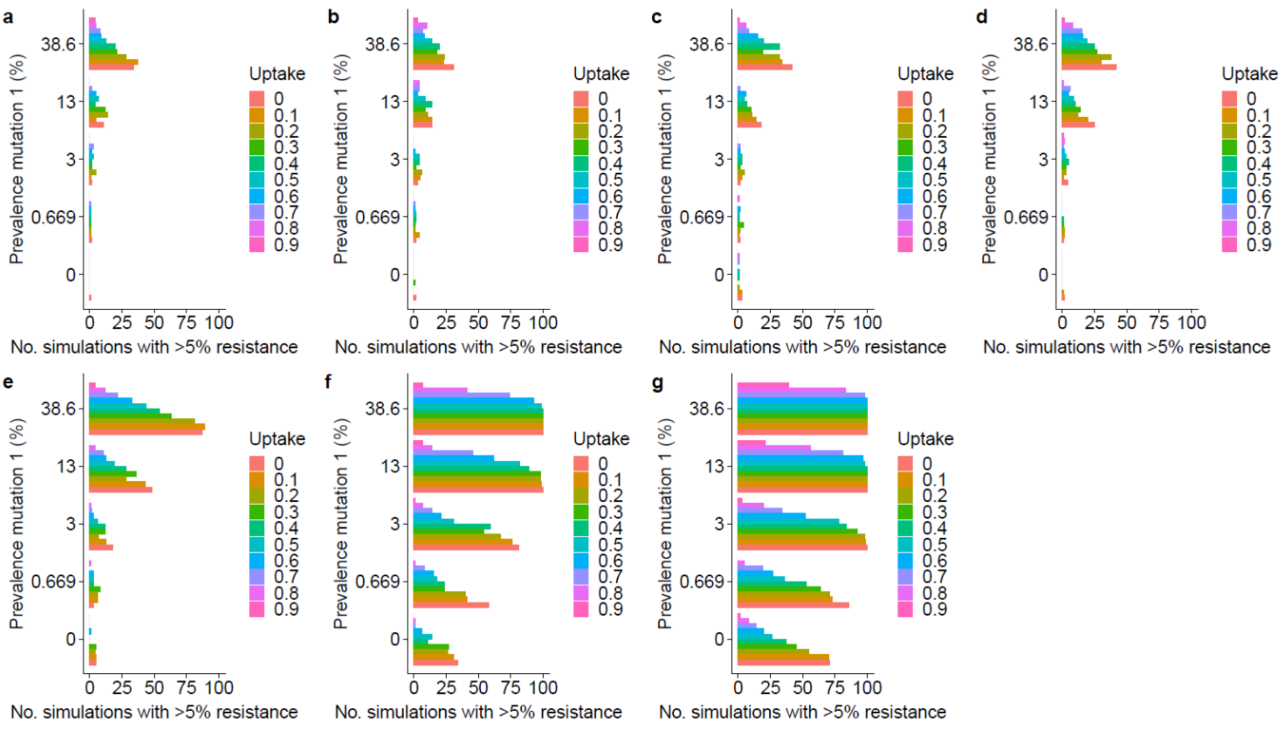

Supplementary Figure 2: Proportion of simulations out of 100 iterations in which the frequency of gepotidacin-resistant strains reaches 5% with different mutation rates, prevalence of ParC D86N and POCT Usage levels. The initial prevalence of GyrA A92T was 1% in all simulations. Mutation rates [substitutions per nucleotide per day]: a) $2.45{\times}10^{-8}$, b) $4.9{\times}10^{-8}$, c) $1.23{\times}10^{-7}$, d) $2.45{\times}10^{-7}$, e) $2.45{\times}10^{-6}$, f) $2.45{\times}10^{-5}$, g) $7.95{\times}10^{-5}$. X-axis: number of simulations out of 100 in which 5% gepotidacin resistance is reached. Y-axis: initial prevalence of ParC D86N.

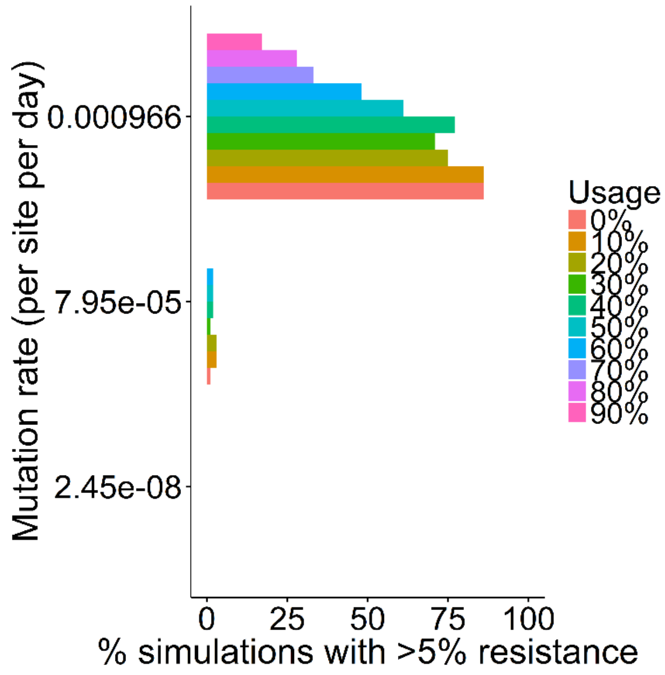

Supplementary Figure 3: Proportion of simulations in which the frequency of gepotidacin-resistant strains reaches 5% with different mutation rates under treatment and POCT Usage levels. Mutation rate without treatment: $2.45{\times}10^{-8}$ substitutions per nucleotide per day, no pre-existing stepping-stone mutations. X-axis: percentage of simulations in which 5% gepotidacin resistance is reached. Y-axis: mutation rate with treatment [substitutions per nucleotide per day].

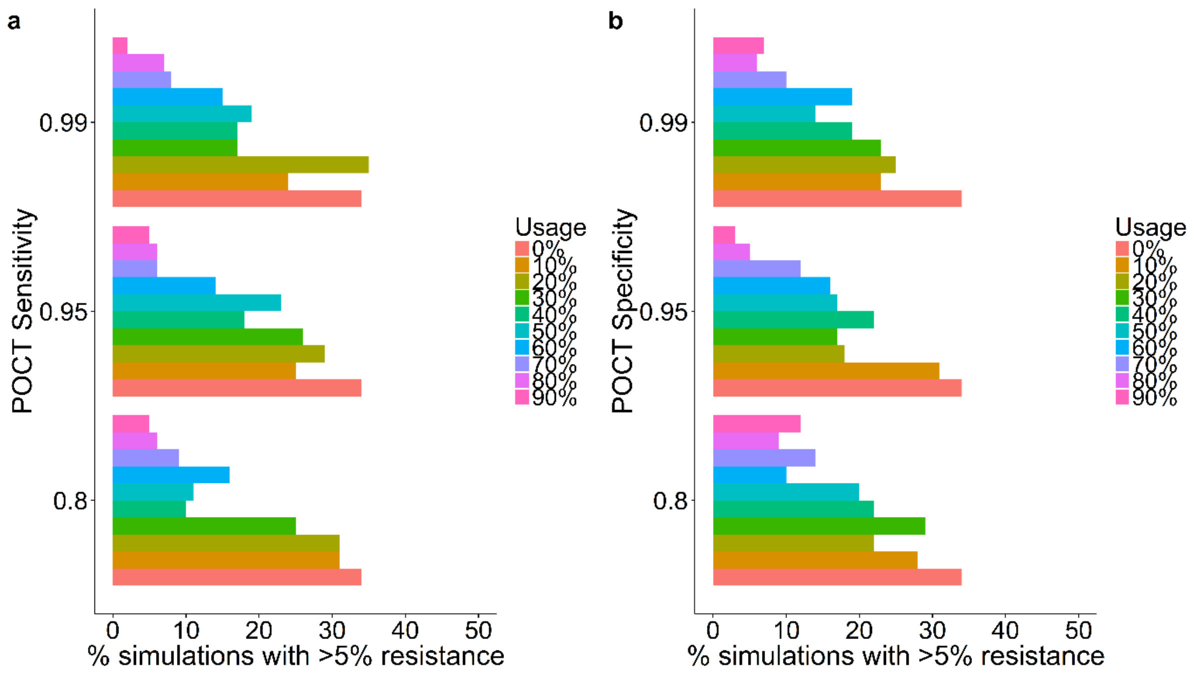

Supplementary Figure 4: Proportion of simulations in which the frequency of gepotidacin-resistant strains reaches 5% for different Usage levels of a POCT. Mutation rate with and without treatment 2.45×10^{-8} substitutions per nucleotide per day. Prevalence of ParC D86N: 38.6%, prevalence of GyrA A92T: 1%. a) Y-axis gives assumed POCT sensitivity, b) Y-axis gives assumed POCT specificity. X-axis: percentage of simulations in which 5% gepotidacin resistance is reached.

Supplementary Text 2 – Deterministic model equations, diagrams and possible state transitions

See Table 2 in main text for explanation of model variables and parameters.

Equations 1: General model of gonorrhoea transmission.

$$\frac{dS}{dt} = -\beta S(I_{00} + I_{10} + I_{01} + I_{11}) + f(I_{00} + I_{10} + I_{01} + I_{11}) + g(T_{00} + T_{10} + T_{01})$$

$$\frac{dI_{00}}{dt} = \beta S I_{00} - f I_{00} - \gamma I_{00} + \sigma_b(I_{10} + I_{01} - 2I_{00})$$

$$\frac{dI_{10}}{dt} = \beta S I_{10} - f I_{10} - \gamma I_{10} + \sigma_b(I_{00} + I_{11} - 2I_{10})$$

$$\frac{dI_{01}}{dt} = \beta S I_{01} - f I_{01} - \gamma I_{01} + \sigma_b(I_{00} + I_{11} - 2I_{01})$$

$$\frac{dI_{11}}{dt} = \beta S I_{11} - f I_{11} - \gamma I_{11} + g T_{11} + \sigma_b(I_{10} + I_{01} - 2I_{11})$$

$$\frac{dT_{00}}{dt} = \gamma I_{00} - g T_{00} - 2\sigma_t T_{00}$$

$$\frac{dT_{10}}{dt} = \gamma I_{10} - g T_{10} + \sigma_t(T_{00} - T_{10})$$

$$\frac{dT_{01}}{dt} = \gamma I_{01} - g T_{01} + \sigma_t(T_{00} - T_{01})$$

$$\frac{dT_{11}}{dt} = \gamma I_{11} - g T_{11} + \sigma_t(T_{10} + T_{01})$$

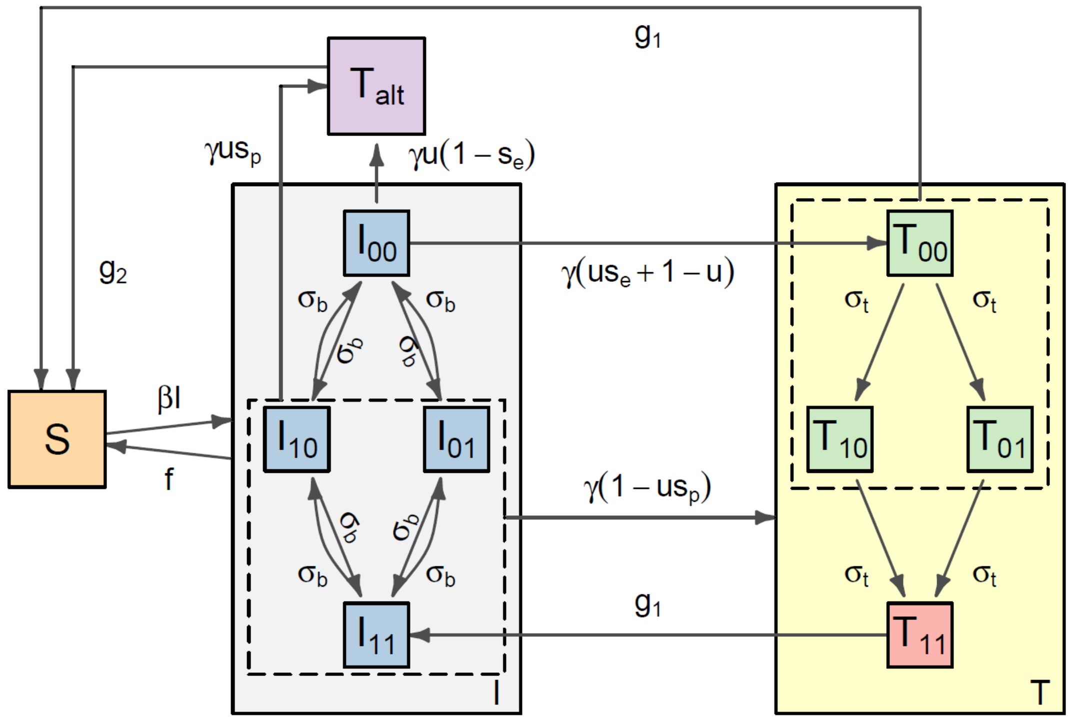

Figure S1: Two-locus gonorrhoea antibiotic resistance model. Point-of-care test (POCT). Gepotidacin is only used for treatment if no resistance mutations are found – otherwise unspecified alternative treatment is used. S – susceptible, I – infected individuals before treatment has been sought, T – infected individuals treated with gepotidacin, T_{alt} – infected individuals treated with alternative antibiotic, subscript 0 – sensitive allele, subscript 1 – resistant allele, β – infection rate parameter, f – recovery rate, γ – treatment rate, g1 – cure rate of gepotidacin, g2 – cure rate of alternative treatment, u – Usage level of POCT, s_e – sensitivity of POCT, s_p – specificity of POCT, σ_b – baseline mutation rate σ_t mutation rate under treatment .

Equations 2: Point-of-care test. See Caption of Figure S1 for explanation of model variables and parameters.

$$\frac{dS}{dt} = -\beta S(I_{00} + I_{10} + I_{01} + I_{11}) + f(I_{00} + I_{10} + I_{01} + I_{11}) + g_1(T_{00} + T_{10} + T_{01}) + \ g_2 T_{alt}$$

$$\frac{dI_{00}}{dt} = \beta S I_{00} - f I_{00} - \ \gamma I_{00} + \ \sigma_b(I_{10} + \ I_{01} - \ 2I_{00})$$

$$\frac{dI_{10}}{dt} = \beta S I_{10} - f I_{10} - \ \gamma I_{10} + \ \sigma_b(I_{00} + I_{11} - \ 2I_{10})$$

$$\frac{dI_{01}}{dt} = \beta S I_{01} - f I_{01} \ - \ \gamma I_{01} + \ \sigma_b(I_{00} + I_{11} - \ 2I_{01})$$

$$\frac{dI_{11}}{dt} = \beta S I_{11} - f I_{11} - \ \gamma I_{11} + g_1 T_{11} + \sigma_b(I_{10} + I_{01} \ - 2I_{11})$$

$$\frac{dT_{00}}{dt} = [us_e\gamma + (1-u)\gamma]I_{00} - g_1T_{00} - 2\sigma_tT_{00}$$

$$\frac{dT_{10}}{dt} = [u(1-s_p)\gamma + (1-u)\gamma]I_{10} - g_1T_{10} + \sigma_t(T_{00} - T_{10})$$

$$\frac{dT_{01}}{dt} = [u(1-s_p)\gamma + (1-u)\gamma]I_{01} - g_1T_{01} + \sigma_t(T_{00} - T_{01})$$

$$\frac{dT_{11}}{dt} = [u(1-s_p)\gamma + (1-u)\gamma]I_{11} - g_1T_{11} + \sigma_t(T_{10} + T_{01})$$

$$\frac{dT_{alt}}{dt} = us_p\gamma(I_{10} + I_{01} + I_{11}) + u(1-s_e)\gamma I_{00} - g_2T_{alt}$$

Table S1: Point-of-care test. Possible state transitions in individual-based stochastic model based on the deterministic equations detailed above. See Caption of Figure S1 for explanation of model variables and parameters.

**Current state**	**Possible transitions**
S	S → I_{00}, S → I_{10}, S → I_{01}, S → I_{11}
I_{00}	I_{00} → S, I_{00} → T_{00}, I_{00} → T_{alt}, I_{00} → I_{10}, I_{00} → I_{01}
I_{10}	I_{10} → S, I_{10} → T_{10}, I_{10} → T_{alt}, I_{10} → I_{00}, I_{10} → I_{11}
I_{01}	I_{01} → S, I_{01} → T_{01}, I_{01} → T_{alt} I_{01} → I_{00}, I_{01} → I_{11}
I_{11}	I_{11} → S, I_{11} → T_{11}, I_{11} → T_{alt}, I_{11} → I_{10}, I_{11} → I_{01}
T_{00}	T_{00} → S, T_{00} → T_{10}, T_{00} → T_{01}
T_{10}	T_{10} → S, T_{10} → T_{11}
T_{01}	T_{01} → S, T_{01} → T_{11}
T_{11}	T_{11} → I_{11}
T_{alt}	T_{alt} → S

References

1. Dona V, Low N, Golparian D, Unemo M. Recent advances in the development and use of molecular tests to predict antimicrobial resistance in *Neisseria gonorrhoeae*. Expert Review of Molecular Diagnostics 2017;17(9):845-859.
2. Lindback E, Gharizadeh B, Ataker F, Airell A, Jalal S, Nyren P, et al. DNA gyrase gene in *Neisseria gonorrhoeae* as indicator for resistance to ciprofloxacin and species verification. International Journal of STD & AIDS 2005;16(2):142-7.
3. Scangarella-Oman NE, Hossain M, Dixon PB, Ingraham K, Min S, Tiffany CA, et al. Microbiological analysis from a phase 2 randomized study in adults evaluating single oral doses of gepotidacin in the treatment of uncomplicated urogenital gonorrhea caused by *Neisseria gonorrhoeae*. Antimicrobial Agents and Chemotherapy 2018;62(12): e01221-18.
4. Farrell DJ, Sader HS, Rhomberg PR, Scangarella-Oman NE, Flamm RK. In Vitro Activity of Gepotidacin (GSK2140944) against *Neisseria gonorrhoeae*. Antimicrobial Agents and Chemotherapy 2017;61(3):e02047-16.
5. Whittles LK, White PJ, Didelot X. Estimating the fitness cost and benefit of cefixime resistance in *Neisseria gonorrhoeae* to inform prescription policy: A modelling study. PLOS Medicine 2017;14(10):e1002416.
6. Handsfield HH, Lipman TO, Harnisch JP, Tronca E, Holmes KK. Asymptomatic gonorrhea in men: Diagnosis, natural course, prevalence and significance. New England Journal of Medicine. 1974;290(3):117-23.
7. Wallin J, Siegel MS. Pharyngeal *Neisseria gonorrhoeae*: coloniser or pathogen? British Medical Journal 1979;1(6176):1462-3.
8. Swinton J, Garnett GP, Brunham RC, Anderson RM. Gonococcal infection, infertility, and population growth: I. Endemic states in behaviourally homogeneous growing populations. IMA Journal of Mathematics Appllied in Medicine & Biology. 1992;9:102-26.
9. Garnett GP, Mertz KJ, Finelli L, Levine WC, St Louis ME. The transmission dynamics of gonorrhoea: modelling the reported behaviour of infected patients from Newark, New Jersey. Philosophical Transactions of the Royal Society of London Series B, Biological Sciences. 1999;354(1384):787-97.
10. Jin F, Prestage GP, Mao L, Kippax SC, Pell CM, Donovan B, et al. Incidence and risk factors for urethral and anal gonorrhoea and chlamydia in a cohort of HIV-negative homosexual men: the Health in Men Study. Sexually Transmitted Infections 2007;83(2):113-9.
11. Johnson LF, Alkema L, Dorrington RE. A Bayesian approach to uncertainty analysis of sexually transmitted infection models. Sexually Transmitted Infections 2010;86(3):169-74.
12. Didelot X, Dordel J, Whittles LK, Collins C, Bilek N, Bishop CJ, et al. Genomic analysis and comparison of two gonorrhea outbreaks. mBio 2016;7(3).
13. Hook EW, 3rd, Richey CM, Leone P, Bolan G, Spalding C, Henry K, et al. Delayed presentation to clinics for sexually transmitted diseases by symptomatic patients. A potential contributor to continuing STD morbidity. Sexually Transmitted Diseases 1997;24(8):443-8.
14. Flynn C, Anschuetz G, Asbel L, Madera R, Johnson CC. Influence of insurance status and demographic features on recognition of symptomatic and asymptomatic gonorrhea cases. Sexually Transmitted Diseases 2015;42(8):419-21.
15. Hossain M, Tiffany CA, Day L, Dumont EF. Population pharmacokinetic modeling of first-time-in-human data of GSK2140944, a novel antimicrobial compound. Abstract of the 53rd Interscientific Conference on Antimicrobial Agents and Chemotherapy, abstract F-1217; 2013.
16. Taylor SN, Morris DH, Avery AK, Workowski KA, Batteiger BE, Tiffany CA, et al. Gepotidacin for the Treatment of Uncomplicated Urogenital Gonorrhea: A Phase 2, Randomized, Dose-Ranging, Single-Oral Dose Evaluation. Clinical Infectious Diseases 2018;67(4):504-12.
17. Höffken G, Lode H, Prinzing K, Borner K, Koeppe P. Pharmacokinetics of ciprofloxacin after oral and parenteral administration. Antimicrobial Agents and Chemotherapy 1985;27(3):375-9.

18. Grad YH, Kirkcaldy RD, Trees D, Dordel J, Harris SR, Goldstein E, et al. Genomic epidemiology of *Neisseria gonorrhoeae* with reduced susceptibility to cefixime in the USA: a retrospective observational study. Lancet Infectious Diseases 2014;14(3):220-6.
19. Ezewudo MN, Joseph SJ, Castillo-Ramirez S, Dean D, Del Rio C, Didelot X, et al. Population structure of *Neisseria gonorrhoeae* based on whole genome data and its relationship with antibiotic resistance. PeerJ 2015;3:e806.
20. Gillespie T, Masterton RG. Investigation into the selection frequency of resistant mutants and the bacterial kill rate by levofloxacin and ciprofloxacin in non-mucoid *Pseudomonas aeruginosa* isolates from cystic fibrosis patients. International Journal of Antimicrobial Agents 2002;19(5):377-82.
21. Gillespie SH, Basu S, Dickens AL, O'Sullivan DM, McHugh TD. Effect of subinhibitory concentrations of ciprofloxacin on *Mycobacterium fortuitum* mutation rates. Journal of Antimicrobial Chemotherapy 2005;56(2):344-8.
22. Gillespie SH, Voelker LL, Ambler JE, Traini C, Dickens A. Fluoroquinolone resistance in *Streptococcus pneumoniae*: evidence that *gyrA* mutations arise at a lower rate and that mutation in *gyrA* or *parC* predisposes to further mutation. Microbial Drug Resistance 2003;9(1):17-24.
23. Han J, Sahin O, Barton YW, Zhang Q. Key role of Mfd in the development of fluoroquinolone resistance in *Campylobacter jejuni*. PLOS Pathogens 2008;4(6):e1000083.
24. Long H, Miller SF, Strauss C, Zhao C, Cheng L, Ye Z, et al. Antibiotic treatment enhances the genome-wide mutation rate of target cells. Proceedings of the National Academy of Sciences of the United States of America 2016;113(18):E2498-505.
25. Martinez JL, Baquero F. Mutation frequencies and antibiotic resistance. Antimicrobial Agents and Chemotherapy. 2000;44(7):1771-7.
26. Brittain C, Childs M, Duley L, Harding J, Hepburn T, Meakin G, et al. Gentamicin versus ceftriaxone for the treatment of gonorrhoea (G-TOG trial): study protocol for a randomised trial. Trials 2016;17(1):558.
27. Nagel M, Reuter T, Jansen A, Szekat C, Bierbaum G. Influence of ciprofloxacin and vancomycin on mutation rate and transposition of IS256 in *Staphylococcus aureus*. International Journal of Medical Microbiology 2011;301(3):229-36.
28. Plasencia V, Borrell N, Macia MD, Moya B, Perez JL, Oliver A. Influence of high mutation rates on the mechanisms and dynamics of *in vitro* and *in vivo* resistance development to single or combined antipseudomonal agents. Antimicrobial Agents & Chemotherapy 2007;51(7):2574-81.
29. Dewi BE, Akira S, Hayashi H, Ba-Thein W. High occurrence of simultaneous mutations in target enzymes and MtrRCDE efflux system in quinolone-resistant *Neisseria gonorrhoeae*. Sexually Transmitted Diseases 2004;31(6):353-9.
30. Saika T, Kobayashi I, Inoue M. A comparison of the microbiological characteristics of *Neisseria gonorrhoeae* isolated from male and female patients with gonorrhea. Chemotherapy 2004;50(2):92-7.
31. Kulkarni S, Bala M, Sane S, Pandey S, Bhattacharya J, Risbud A. Mutations in the *gyrA* and *parC* genes of quinolone-resistant *Neisseria gonorrhoeae* isolates in India. International Journal of Antimicrobial Agents 2012;40(6):549-53.
32. Allen VG, Farrell DJ, Rebbapragada A, Tan J, Tijet N, Perusini SJ, et al. Molecular analysis of antimicrobial resistance mechanisms in *Neisseria gonorrhoeae* isolates from Ontario, Canada. Antimicrobial Agents and Chemotherapy 2011;55(2):703-12.
33. Sethi S, Golparian D, Bala M, Dorji D, Ibrahim M, Jabeen K, et al. Antimicrobial susceptibility and genetic characteristics of *Neisseria gonorrhoeae* isolates from India, Pakistan and Bhutan in 2007-2011. BMC Infectious Diseases2013;13:35.
34. European Centre for Disease Prevention and Control. Gonococcal antimicrobial susceptibility surveillance in Europe. ECDC Surveillance Report 2014.

35. Centers for Disease Control and Prevention. Sexually transmitted disease surveillance 2014: Gonococcal Isolate Surveillance Project (GISP). Atlanta: US Department of Health and Human Services 2016.
36. Public Health Agency of Canada. National surveillance of antimicrobial susceptibilities of *Neisseria gonorrhoeae* annual summary 2014. Ministry of Health Canada 2015.
37. Wi T, Lahra MM, Ndowa F, Bala M, Dillon JR, Ramon-Pardo P, et al. Antimicrobial resistance in *Neisseria gonorrhoeae*: Global surveillance and a call for international collaborative action. PLOS Medicine 2017;14(7):e1002344.
38. Tiejun Z, Xiaoming Z, Jilun Z, Yinghu Z, Yanhua R, Yue C, et al. Fluoroquinolone resistance among *Neisseria gonorrhoeae* isolates from Shanghai, China: detection of quinolone resistance-determining region mutations. Indian Journal of Medical Research 2009;129(6):701-6.
39. Grad YH, Harris SR, Kirkcaldy RD, Green AG, Marks DS, Bentley SD, et al. Genomic epidemiology of gonococcal resistance to extended-spectrum cephalosporins, macrolides, and fluoroquinolones in the United States, 2000-2013. J Infect Dis 2016;214(10):1579-87.
40. Demczuk W, Lynch T, Martin I, Van Domselaar G, Graham M, Bharat A, et al. Whole-genome phylogenomic heterogeneity of *Neisseria gonorrhoeae* isolates with decreased cephalosporin susceptibility collected in Canada between 1989 and 2013. Journal of Clinical Microbiology 2015;53(1):191-200.
41. Demczuk W, Martin I, Peterson S, Bharat A, Van Domselaar G, Graham M, et al. Genomic epidemiology and molecular resistance mechanisms of azithromycin-resistant *Neisseria gonorrhoeae* in Canada from 1997 to 2014. Journal of Clinical Microbiology 2016;54(5):1304-13.
42. De Silva D, Peters J, Cole K, Cole MJ, Cresswell F, Dean G, et al. Whole-genome sequencing to determine transmission of *Neisseria gonorrhoeae*: an observational study. Lancet Infectious Diseases 2016;16(11):1295-303.
43. Eyre DW, De Silva D, Cole K, Peters J, Cole MJ, Grad YH, et al. WGS to predict antibiotic MICs for *Neisseria gonorrhoeae*. Journal of Antimicrobial Chemotherapy 2017: 72(7):1937-1947.
44. Kwong JC, Chow EPF, Stevens K, Stinear TP, Seemann T, Fairley CK, et al. Whole-genome sequencing reveals transmission of gonococcal antibiotic resistance among men who have sex with men: an observational study. Sexually Transmitted Infections 2018;94(2):151-7.
45. Buckley C, Forde BM, Trembizki E, Lahra MM, Beatson SA, Whiley DM. Use of whole genome sequencing to investigate an increase in *Neisseria gonorrhoeae* infection among women in urban areas of Australia. Scientific Reports 2018;8(1):1503.
46. Cehovin A, Harrison OB, Lewis SB, Ward PN, Ngetsa C, Graham SM, et al. Identification of novel *Neisseria gonorrhoeae* lineages harboring resistance plasmids in coastal Kenya. Journal of Infectious Diseases 2018;218(5):801-8.
47. Fifer H, Cole M, Hughes G, Padfield S, Smolarchuk C, Woodford N, et al. Sustained transmission of high-level azithromycin-resistant *Neisseria gonorrhoeae* in England: an observational study. Lancet Infectious Diseases 2018;18(5):573-81.
48. Harris SR, Cole MJ, Spiteri G, Sánchez-Busó L, Golparian D, Jacobsson S, et al. Public health surveillance of multidrug-resistant clones of *Neisseria gonorrhoeae* in Europe: a genomic survey. Lancet Infectious Diseases 2018;18(7): 758-768.
49. Lee RS, Seemann T, Heffernan H, Kwong JC, Goncalves da Silva A, Carter GP, et al. Genomic epidemiology and antimicrobial resistance of *Neisseria gonorrhoeae* in New Zealand. Journal of Antimicrobial Chemotherapy 2018;73(2):353-64.
50. Ryan L, Golparian D, Fennelly N, Rose L, Walsh P, Lawlor B, et al. Antimicrobial resistance and molecular epidemiology using whole-genome sequencing of *Neisseria gonorrhoeae* in Ireland, 2014-2016: focus on extended-spectrum cephalosporins and azithromycin. European Journal of Clinical Microbiology & Infectious Diseases 2018;37(9):1661-72.
51. Sánchez-Busó L, Golparian D, Corander J, Grad YH, Ohnishi M, Flemming R, et al. Antimicrobial exposure in sexual networks drives divergent evolution in modern gonococci. bioRxiv 2018(334847).

52. Sánchez-Busó L, Golparian D, Corander J, Grad YH, Ohnishi M, Flemming R, et al. The impact of antimicrobials on gonococcal evolution. Nature Microbiology. 2019;4(11):1941-50.
53. Yahara K, Nakayama SI, Shimuta K, Lee KI, Morita M, Kawahata T, et al. Genomic surveillance of *Neisseria gonorrhoeae* to investigate the distribution and evolution of antimicrobial-resistance determinants and lineages. Microbial Genomics 2018;4(8):e000205.
54. Peng JP, Yin YP, Chen SC, Yang J, Dai XQ, Zheng HP, et al. A whole-genome sequencing analysis of *Neisseria gonorrhoeae* isolates in China: An observational study. EClinicalMedicine 2019;7:47-54.
55. Thomas JC, Seby S, Abrams AJ, Cartee J, Lucking S, Vidyaprakash E, et al. Evidence of recent genomic evolution in gonococcal strains with decreased susceptibility to cephalosporins or azithromycin in the United States, 2014-2016. Journal of Infectious Diseases 2019;220(2):294-305.
56. Williamson DA, Chow EPF, Gorrie CL, Seemann T, Ingle DJ, Higgins N, et al. Bridging of *Neisseria gonorrhoeae* lineages across sexual networks in the HIV pre-exposure prophylaxis era. Nature Communications 2019;10(1):3988.
57. Lan PT, Golparian D, Ringlander J, Van Hung L, Van Thuong N, Unemo M. Genomic analysis and antimicrobial resistance of *Neisseria gonorrhoeae* isolates from Vietnam in 2011 and 2015-16. Journal of Antimicrobial Chemotherapy 2020; 75(6):1432-1438.
58. Town K, Bolt H, Croxford S, Cole M, Harris S, Field N, et al. *Neisseria gonorrhoeae* molecular typing for understanding sexual networks and antimicrobial resistance transmission: A systematic review. Journal of Infectious Diseases 2018;76(6):507-14.
59. Kohler T, Michea-Hamzehpour M, Plesiat P, Kahr AL, Pechere JC. Differential selection of multidrug efflux systems by quinolones in *Pseudomonas aeruginosa*. Antimicrobial Agents and Chemotherapy 1997;41(11):2540-3.
60. Mo CY, Manning SA, Roggiani M, Culyba MJ, Samuels AN, Sniegowski PD, et al. Systematically altering bacterial SOS activity under stress reveals therapeutic strategies for potentiating antibiotics. mSphere 2016;1(4):e0163-16.
